# Psychrobacter saeujeotis sp. nov., a novel halophilic bacterium isolated from salted shrimp jeotgal

**DOI:** 10.1099/ijsem.0.006734

**Published:** 2025-03-21

**Authors:** Eiseul Kim, Yinhua Cai, Seung-Min Yang, Woojung Lee, Hae-Yeong Kim

**Affiliations:** 1 Institute of Life Sciences & Resources and Department of Food Science and Biotechnology, Kyung Hee University, Yongin 17104, Republic of Korea

**Keywords:** marine species, novel species, *Psychrobacter saeujeotis*, salted fermented shrimp

## Abstract

A novel bacterial species, designated FBL11^T^, was isolated from salted shrimp jeotgal, a traditional Korean fermented food sampled in the Republic of Korea. This strain showed the highest 16S rRNA gene sequence similarity to *Psychrobacter halodurans* strain F2608^T^ (NR_181578.1) at 98.3%. The genome size of strain FBL11^T^ was 3,294,493 bp with a G+C content of 42.5 mol%. Computation of relatedness indicates that strain FBL11^T^ shares the highest relatedness of 78.5% with *Psychrobacter fulvigenes* strain KC-40^T^ (NZ_CAJGZP01) and 25.2% with *Psychrobacter piechaudii* strain CIP110854^T^ (NZ_FUGE01), with values clearly below the cut-offs for species distinction. Polyphasic characterization using biochemical tests and matrix-assisted laser desorption/ionization time-of-flight MS analysis confirmed these findings. Strain FBL11^T^ grew at 10–30 °C (optimum, 20–30 °C) with 0–15% NaCl (w/v; optimum, 3–6%). Analysis of biosynthetic gene clusters responsible for secondary metabolite production revealed that strain FBL11^T^ generates unique products such as beta-lactone and redox-cofactors within this genus. Based on the genomic and phenotypic data obtained, we propose that strain FBL11^T^ represents a novel species, for which we propose the name *Psychrobacter saeujeotis* sp. nov. (type strain FBL11^T^=KACC 23745^T^=KCTC 8655^T^=JCM 37231^T^).

## Introduction

The genus *Psychrobacter*, first described in 1986 by Juni and Heym [1], comprises over 46 species with officially recognized names, according to the List of Prokaryotic Names with Standing in Nomenclature (https://lpsn.dsmz.de/genus/psychrobacter). These species are capable of thriving in cold or saline environments, displaying psychrophilic, psychrotolerant and halotolerant traits [2,3]. The genus *Psychrobacter* is commonly found in diverse environments. Notably, *Psychrobacter* species have been isolated from various unique habitats, including marine crustaceans [4], pigeon faeces [5], deep and surface ice [6], Antarctic soils enriched by bird presence [7], penguins [4] and white storks [8]. *Psychrobacter* species have also been discovered in frozen soils and permafrost in Siberia and Antarctica [9,10]. While most *Psychrobacter* strains are cold-adapted [11], there are also mesophilic strains [7]. The diverse range of habitats where *Psychrobacter* is found offers a unique opportunity to study habitat-specific adaptations while minimizing phylogenetic influences [9,12]. In particular, *Psychrobacter* is known for its production of various bioactive compounds, including antibiotics [13], antifungals [14] and anticancer agents [15].

Typically, members of the genus *Psychrobacter* are Gram-negative and can take the form of coccoid or rod-shaped cells. They are non-motile, oxidase-positive bacteria that are tolerant to both cold and saline conditions [16]. Their DNA typically contains a G+C content ranging from 41 to 50 mol%. Notably, *Psychrobacter marincola* produces capsular polysaccharides containing 5,7-di-*N*-acetyl-pseudaminic acid, but this feature has not been observed across the entire genus [15]. The phylogenetic analysis involves various genomic methods such as analysing the 16S rRNA gene sequence, digital DNA–DNA hybridization (dDDH) and average nucleotide identity (ANI) [17]. In this study, we conducted phylogenetic and biochemical analyses to characterize strain FBL11^T^. Based on our findings, we propose that strain FBL11^T^ represents a new species within the genus *Psychrobacter*, which we designate as *Psychrobacter saeujeotis* sp. nov.

## Isolation and ecology

The strain FBL11^T^ was isolated from salted shrimp jeotgal, a traditional Korean fermented food, with a salinity of 18–23%, to identify bacteria originating from shrimp that can survive in high-salt conditions. The sample was collected from a local market in Ganggyeong-eup, Korea (36° 09′ 36.5″ N 127° 00′ 37.8″ E), and immediately stored under refrigeration before being transported on ice to the laboratory to ensure its preservation. Upon arrival, it was used promptly for experiments.

To isolate the bacteria, 25 g of salted shrimp jeotgal was mixed with 225 ml of PBS and homogenized. The mixture was then serially diluted in a tenfold series, and 100 µl from appropriate dilutions was spread onto marine agar plates (Difco, Detroit, MI, USA). Marine agar was chosen for its nutrient-rich composition, supporting the growth of salt-tolerant bacteria expected in the high-salt environment of salted shrimp jeotgal. The plates were incubated aerobically at 30 °C for 48 h. This temperature was selected to reflect the upper end of typical fermentation conditions, which occur at room temperature, promoting optimal microbial growth. After incubation, colonies were selected based on their distinct morphological features, such as size, shape, colour and texture, to capture a diverse range of bacteria. These colonies were sub-cultured onto fresh marine agar plates to ensure purity, and a pure culture of strain FBL11^T^ was successfully isolated. For long-term preservation, the strain was stored at −80 °C with 20% (v/v) glycerol. Following retrieval from storage, the strain was passaged once on marine agar before being deposited in the Korean Agricultural Culture Collection (KACC), Korean Collection for Type Cultures (KCTC) and Japan Collection of Microorganisms (JCM) for future studies.

## 16S rRNA gene analysis

Genomic DNA extraction was performed using the DNeasy Blood and Tissue Kit (Qiagen, Hilden, Germany), following the manufacturer’s instructions. The quality and quantity of the extracted DNA were assessed using a NanoDrop spectrophotometer (Thermo Fisher Scientific, Waltham, MA, USA). For the amplification of the 16S rRNA gene, PCR was carried out using bacterial universal primers 27F (5′-AGAGTTTGATCCTGGCTCAG-3′) and 1492R (5′-CTACGGCTACCTTGTTACGA-3′) [18]. The PCR reaction mixture, with a final volume of 25 µl, contained 10 ng of DNA, 10× buffer, 0.2 mM dNTPs, 0.5 U Ex Taq polymerase and 0.4 µM of each primer. The PCR conditions were as follows: initial denaturation at 94 °C for 5 min, followed by 30 cycles of denaturation at 94 °C for 30 s, annealing at 60 °C for 1 min, extension at 72 °C for 30 s and a final extension at 72 °C for 5 min [19]. The PCR product was purified using the QIAquick PCR Purification Kit from Qiagen and then subjected to Sanger capillary electrophoresis sequencing by Bionics (Seoul, Korea).

A 16S rRNA sequence comparison of strain FBL11^T^ was conducted using the EzTaxon database (https://www.ezbiocloud.net/identify), revealing high similarity with several *Psychrobacter* species. Among these, *Psychrobacter halodurans* F2608^T^ (MW405795) showed the highest similarity at 98.07%, followed by *Psychrobacter celer* SW-238^T^ (AY842259) at 97.59% and *Psychrobacter coccoides* F1192^T^ (MW405808) at 97.52%. These findings suggest that strain FBL11^T^ is closely related to members of the *Psychrobacter* genus, with similarities reaching up to 98.07%.

**Fig. 1.**
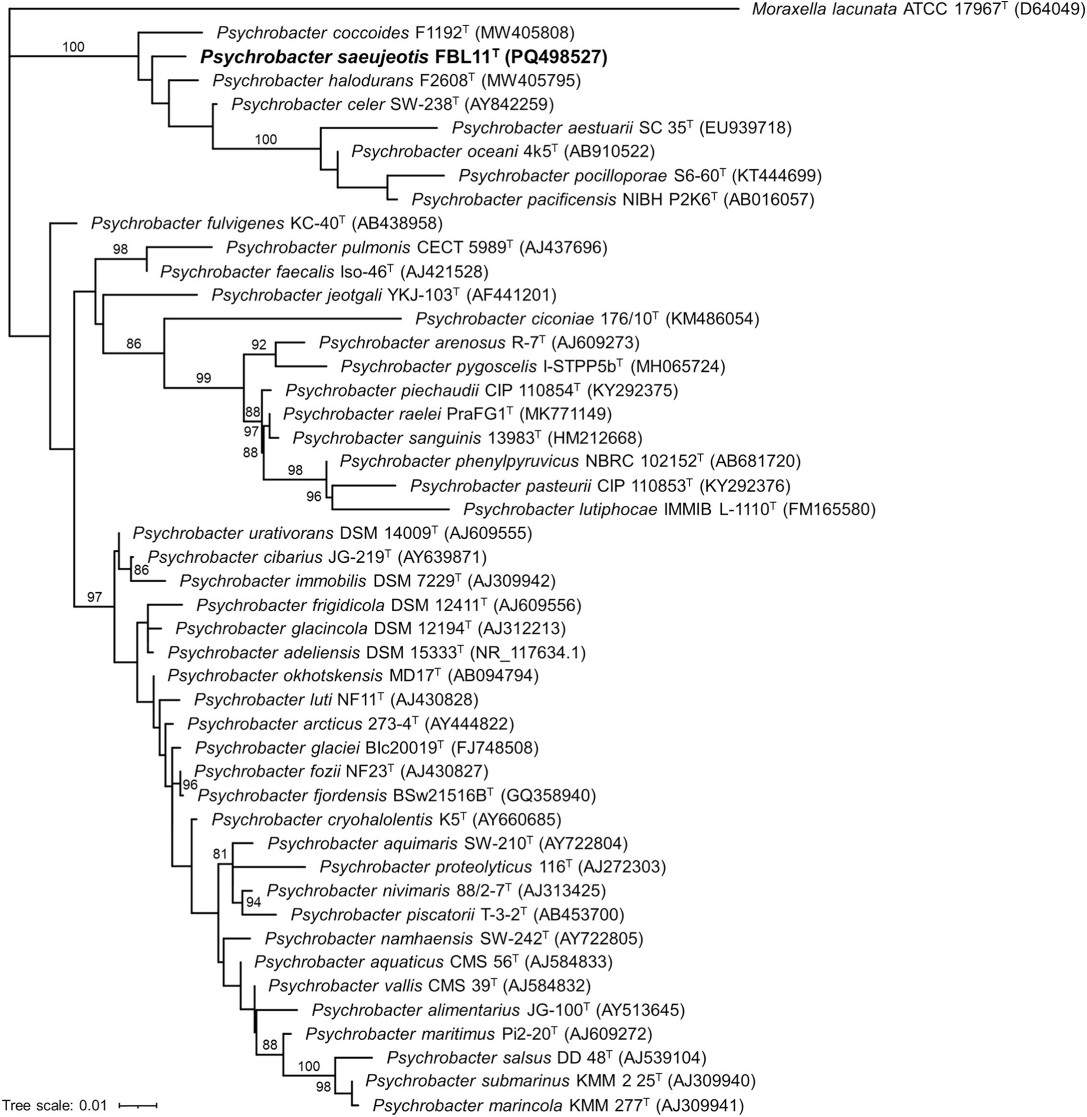
Maximum likelihood phylogenetic tree based on 16S rRNA gene sequences, reconstructed using the TPM3+I+R2 evolutionary model, illustrating the relationships between strain FBL11^T^ and other *Psychrobacter* species. *Moraxella lacunata* was used as the outgroup. Bootstrap values over 80% (from 1,000 replicates) are shown at each node, with the species from this study marked in bold. The scale bar indicates 1% substitutions per site.

A phylogenetic analysis was conducted using the 16S rRNA gene sequence of strain FBL11^T^, together with sequences from 45 type strains of *Psychrobacter* species with validly published names as of 20 October 2024 (Table S1, available in the online Supplementary Material). Since the 16S rRNA gene sequence of *Psychrobacter communis* is unavailable, it was omitted from the analysis. Sequence alignment was performed in clustal omega [20] with default settings, and details of nucleotide sequence lengths are listed in Table S2. To ensure accurate phylogenetic inference, the best-fit evolutionary model was determined using ModelFinder in IQ-TREE version 2.3.6 [21], with model selection based on the Bayesian information criterion (BIC). The optimal model, TPM3+I+R2, which accounts for invariant sites and rate heterogeneity, was confirmed as the best fit according to BIC (Table S3). This model was then used to construct a maximum likelihood phylogenetic tree, revealing that strain FBL11^T^ clusters closely with *P. coccoides* F1192^T^ (MW405808), *P. halodurans* F2608^T^ (MW405795) and *P. celer* SW-238^T^ (AY842259) (Fig. 1). The neighbour-joining method produced a similar clustering pattern, further supporting the close relationship of strain FBL11^T^ with these three species (Fig. S1).

## 23S rRNA and *gyrB* gene analysis

In this study, complementary analyses on the 23S rRNA and *gyrB* genes were conducted to enhance the phylogenetic assessment alongside the 16S rRNA gene analysis. Genomes for *Psychrobacter* type strains were downloaded from the National Center for Biotechnology Information (NCBI) database (Table S4), and the 23S rRNA and *gyrB* gene sequences were extracted using Python script. After alignment with clustal omega, phylogenetic trees were generated using the maximum likelihood method in IQ-TREE version 2.3.6, selecting the best-fit evolutionary model using ModelFinder to further support the observed phylogenetic relationships.

For the phylogenetic analysis of the 23S rRNA gene, the best-fit model was determined to be GTR+F+R2 according to the BIC. A total of 42 strains were analysed, including the outgroup *Moraxella lacunata* CCUG 4441^T^, 40 *Psychrobacter* type strains and strain FBL11^T^. Strains with shorter 23S rRNA gene sequences, specifically those under 1,687 bp, were excluded from the analysis, as most other genomes contained sequences over 2,100 bp. For the *gyrB* gene analysis, the TNe+I+R4 model was selected as the best fit according to BIC. This analysis included a total of 47 strains. Due to its comparatively short *gyrB* gene sequence, *Psychrobacter aestuarii* SC 35^T^ was excluded to maintain consistency and accuracy in sequence length across the dataset.

Phylogenetic analyses based on the 23S and *gyrB* gene sequences positioned strain FBL11^T^ in close proximity to species such as *P. coccodies* F1192^T^ and *P. halodurans* F2608^T^. In the 23S gene tree, FBL11^T^ clustered clearly with *P. coccodies* F1192^T^ (Fig. S2), whereas the *gyrB* gene analysis positioned it closer to *P. halodurans* F2608^T^ and *P. celer* DSM 23510^T^ (Fig. S3). In comparison, the 16S rRNA gene analysis consistently grouped FBL11^T^ with similar *Psychrobacter* species and provided the most stable placement near *P. coccodies* F1192^T^ and *P. halodurans* F2608^T^. These results confirm that strain FBL11^T^ belongs to the genus *Psychrobacter*. The consistent clustering of FBL11^T^ with species, such as *P. coccodies* F1192^T^ and *P. halodurans* F2608^T^ across multiple genetic markers, including the 23S, *gyrB*, and 16S rRNA gene, reinforces its phylogenetic placement within the *Psychrobacter* genus, offering support for its classification.

## Genome features

The genomic DNA extracted from the strain FBL11^T^ was subjected to paired-end sequencing using Illumina MiSeq (Chicago, IL, USA). The raw reads were then *de novo* assembled using CLC Workbench version 12.0 (Qiagen). Whole-genome assemblies of strain FBL11^T^ were compared with those of 46 *Psychrobacter* type strains obtained from the NCBI genome database. Genomic comparisons were performed using ANI and dDDH to assess the genetic relatedness between the strains [22]. Detailed information on genome availability, nomenclature and taxonomic status for each *Psychrobacter* strain available in the databases is provided in Tables S1 and S4. The assemblies for strain FBL11^T^ were annotated using the Prokaryotic Genome Annotation Pipeline version 6.7 from NCBI [23].

The assembled genome sequence of strain FBL11^T^ is 3,294,493 bp, composed of 84 contigs with an N_50_ of 992,491 bp and a G+C content of 42.5 mol%. This genome encodes 2,864 coding genes, 42 tRNAs, and 3 rRNAs. For comparison, the closest phylogenetic relative, *Psychrobacter fulvigenes* strain KC-40^T^, has a genome size of 3,465,773 bp and a G+C content of 44.1 mol%. Although *P. fulvigenes* KC-40^T^ did not exhibit high similarity in the 16S rRNA gene analysis, it was the closest relative in the whole-genome sequencing analysis. This discrepancy is likely because 16S rRNA gene analysis examines a single conserved region, which may not fully capture genomic diversity, whereas whole-genome sequencing offers a more comprehensive comparison.

ANI was calculated using whole genome assemblies with the ANI calculator (https://www.ezbiocloud.net/tools/ani), and strain FBL11^T^ shared the highest ANI value (78.5%) with *P. fulvigenes* strain KC-40^T^ (NZ_CAJGZP01) (Table 1). These results indicate that the overall genome similarity between strain FBL11^T^ and its closest relatives is below the 95% threshold for species grouping [24]. dDDH analysis was performed using the Type Strain Genome Server (TYGS) (https://tygs.dsmz.de/) by applying formula 2. Strain FBL11^T^ showed the highest dDDH value (25.2%) against *Psychrobacter piechaudii* strain CIP110854^T^ (NZ_FUGE01), which is below the 70% threshold for species delineation [25]. The TYGS results further confirm that strain FBL11^T^ represents a new species within the *Psychrobacter* genus. This conclusion is supported by closely related type strains identified through the MASH algorithm, phylogenetic inference using the balanced minimum evolution method and pairwise genome comparisons via dDDH. Additionally, the distinct G+C content differentiates FBL11^T^ from other *Psychrobacter* species, supporting its classification as a novel species. To justify the classification of strain FBL11^T^ as a new species, the established criteria for species delineation were applied: 16S rRNA sequence similarity below 97% [17,26], dDDH values under 70% [25] and ANI values under 95% [24]. Strain FBL11^T^ met these thresholds, reinforcing its classification as a novel species within the *Psychrobacter* genus.

**Table 1.** ANI and dDDH values (%) between strain FBL11^T^ and type strains of all species within the genus *Psychrobacter*

Strain	Type strain no.	Assembly no.	ANI	dDDH
*Psychrobacter adeliensis*	SJ 14^T^	GCF_904845895.1	77.3	23.1
*P. aestuarii*	SC 35^T^	GCF_904846035.1	73.3	21.5
*Psychrobacter alimentarius*	JG 100^T^	GCF_904845935.1	76.9	21.8
*Psychrobacter aquaticus*	CMS 56^T^	GCF_000471625.1	77.3	21.8
*Psychrobacter aquimaris*	DSM 16329^T^	GCF_016107525.1	77.9	22.6
*Psychrobacter arcticus*	273-4^T^	GCF_000012305.1	77.7	22.6
*Psychrobacter arenosus*	R7^T^	GCF_904848165.1	72.6	22.9
*Psychrobacter celer*	DSM 23510^T^	GCF_016107555.1	78.1	24.1
*Psychrobacter cibarius*	DSM 16327^T^	GCF_016107535.1	77.8	22.2
*Psychrobacter ciconiae*	176-10^T^	GCF_904846055.1	73.0	22.6
*P. coccoides*	F1192^T^	GCF_017498085.1	77.2	21.7
*P. communis*	Sa4CVA2^T^	GCF_014836505.1	77.5	22.3
*Psychrobacter cryohalolentis*	K5^T^	GCF_000013905.1	77.4	22.5
*Psychrobacter faecalis*	Iso-46^T^	GCF_904845915.1	77.4	22.2
*Psychrobacter fjordensis*	BSw21516B^T^	GCF_904845995.1	77.4	22.5
*Psychrobacter fozii*	CECT 5889^T^	GCF_003217155.1	77.4	22.2
*Psychrobacter frigidicola*	ACAM 304^T^	GCF_007997305.1	76.1	21
*P. fulvigenes*	KC-40^T^	GCF_904846155.1	78.5	23.5
*Psychrobacter glaciei*	KCTC 42280^T^	GCF_014652895.1	78.0	22.8
*Psychrobacter glacincola*	ACAM483^T^	GCF_904846215.1	77.6	23
*Psychrobacter halodurans*	F2608^T^	GCF_017498075.1	77.9	23.7
*Psychrobacter immobilis*	DSM 7229^T^	GCF_003148585.1	77.7	22.3
*Psychrobacter jeotgali*	YKJ-103^T^	GCF_904846315.1	76.2	21.8
*Psychrobacter luti*	CECT 5885^T^	GCF_014192115.1	77.9	22.5
*Psychrobacter lutiphocae*	DSM 21542^T^	GCF_000382145.1	71.8	22.1
*Psychrobacter marincola*	KMM 277^T^	GCF_904846325.1	77.3	22.9
*Psychrobacter maritimus*	Pi2-20^T^	GCF_904846295.1	78.1	23.2
*Psychrobacter namhaensis*	DSM 16330^T^	GCF_016107545.1	77.4	22.2
*Psychrobacter nivimaris*	88/2-7^T^	GCF_904846365.1	77.5	22.7
*Psychrobacter oceani*	4k5^T^	GCF_904846375.1	77.2	22.5
*Psychrobacter okhotskensis*	MD 17^T^	GCF_904846405.1	78.4	23.6
*Psychrobacter pacificensis*	NBRC 103191^T^	GCF_030160475.1	77.2	22.6
*Psychrobacter pasteurii*	CIP 110853^T^	GCF_900162815.1	72.0	23.3
*Psychrobacter phenylpyruvicus*	DSM 7000^T^	GCF_000685805.1	71.5	21.7
*P. piechaudii*	CIP 110854^T^	GCF_900162825.1	71.9	25.2
*Psychrobacter piscatorii*	T-3-2^T^	GCF_904846415.1	77.3	22.2
*Psychrobacter pocilloporae*	S6-60^T^	GCF_029872915.1	77.2	23.1
*Psychrobacter proteolyticus*	116^T^	GCF_904846455.1	77.3	22.8
*Psychrobacter pulmonis*	S-606^T^	GCF_904846465.1	77.3	22.1
*Psychrobacter pygoscelis*	I-STPP5b^T^	GCF_004335015.1	72.8	22.5
*Psychrobacter raelei*	PraFG1^T^	GCF_022631235.3	72.4	25.6
*Psychrobacter salsus*	DD48^T^	GCF_904846445.1	76.0	22.5
*Psychrobacter sanguinis*	13983^T^	GCF_904846515.1	71.9	24.2
*Psychrobacter submarinus*	KMM 225^T^	GCF_904846685.1	75.9	21.9
*Psychrobacter urativorans*	ACAM534^T^	GCF_904846695.1	75.8	22.4
*Psychrobacter vallis*	CMS 39^T^	GCF_904846715.1	77.4	22.2

Moreover, antiSMASH version 7 analysis identified two biosynthetic gene clusters related to secondary metabolite production in strain FBL11^T^, specifically beta-lactone and redox-cofactors. While these clusters are also present in other *Psychrobacter* species, beta-lactone biosynthetic gene clusters are found in all, but 4 type strains, and redox-cofactor gene clusters are found in 19 type strains. Their presence in strain FBL11^T^ highlights its unique metabolic capabilities. However, these features are more reflective of the strain’s functional characteristics rather than a critical factor for its taxonomic classification.

## Physiology

For phenotypic and comparative analysis, strain FBL11^T^ was routinely cultured on marine agar plates and incubated aerobically at 30 °C for 48 h. Its growth was also evaluated on nutrient agar (NA) (Difco) and trypticase soya agar (Difco), with growth observed after 48 h of incubation on both media. However, the most robust growth occurred on marine agar plates, where the strain exhibited the highest growth rate and colony density. This enhanced growth can be attributed to the nutrient composition of marine agar, which contains higher concentrations of salts such as sodium chloride, magnesium chloride and calcium chloride, better suited to the growth requirements of marine-associated bacteria like *Psychrobacter*.

To determine the optimal growth temperature, the strain was incubated on agar plates at temperatures ranging from 0 to 37 °C (0, 5, 10, 15, 20, 25, 30 and 37 °C). The best growth was observed between 10 and 30 °C, with the optimal range identified as 20–30 °C. To determine a more specific optimal temperature, we conducted additional experiments within the 20–30 °C range and performed statistical analysis on the cultured strains. The results revealed significant differences (*P*=2e−16) in strain counts at 20, 25 and 30 °C, leading us to identify 30 °C as the optimal temperature range for the growth of strain FBL11^T^. The tolerance of strain FBL11^T^ to varying pH levels and NaCl concentrations was tested on NA. pH levels ranged from 4.0 to 8.0 (adjusted in one-unit intervals using 1 N HCl and 1 N NaOH), and NaCl concentrations ranged from 0 to 30% (0, 3, 6, 9, 12, 15, 18, 21, 24, 27 and 30% w/v). Optimal growth (highest growth rate and colony density) occurred with NaCl concentrations of 0–15%, with weak growth (reduced colony density and slower growth) observed at 18–21% NaCl and no growth observed at 24–30% NaCl, across a pH range of 6.0–8.0.

Substrate and enzymatic reactions were assessed using the API20NE and APIZYM systems following the manufacturer’s instructions (bioMérieux, Marcy-l'Étoile, France), except for the incubation temperature, which was set at 30 °C. The API AUX medium recommended for Gram-negative bacteria was utilized for the API20NE kit. The main characteristics distinguishing strain FBL11^T^ from its closest related species are summarized in Table 2. Results from the API 20NE system indicated positive urease and cytochrome oxidase activities, along with the assimilation of trisodium citrate. Additionally, nitrate reduction to nitrites was observed, while unmentioned reactions remained negative during the incubation period. In the API ZYM system, reactions were observed for alkaline and acid phosphatase, esterase (C4), esterase lipase (C8), leucine arylamidase, naphthol-AS-BI-phosphohydrolase and *N*-acetyl-*β*-glucosaminidase. Specifically, compared with four other closely related species (*P. celer* JCM 12601^T^, *P. piechaudii* CIP110854^T^, *P. fulvigenes* KC-40^T^, *P. coccoides* F1192^T^ and *P. halodurans* F2608^T^), the esterase lipase (C8) reaction was uniquely observed in strain FBL11^T^. No reactions were observed for substrates including lipase (C14), valine arylamidase, cystine arylamidase, trypsin, *α*-chymotrypsin, acid phosphatase, *α*-galactosidase, *β*-glucuronidase, *α*- and *β*-glucosidase, *α*-mannosidase and *α*-fucosidase.

**Table 2.** Differential phenotypic characteristics between strain FBL11^T^ and type strains 1, FBL11^T^; 2, *P. celer* JCM 12601^T^ [32]; 3, *P. piechaudii* CIP110854^T^ [29]; 4, *P. fulvigenes* KC-40^T^ [4]; 5, *P. coccoides* F1192^T^ [32]; 6, *P. halodurans* F2608^T^ [32].

Characteristic	1	2	3	4	5	6
Temperature range (optimum) for growth (°C)	20–30	25–30	30	25–28	30	30–33
Salt tolerance range for growth (%)	0–15	0–16	0–15	0–12	0–12	0.5–12
Optimum	3–6	2–3	na	na	3–4	4–5
pH range (optimum) for growth	7.0	7.0–8.0	na	na	7.0–7.5	6.5–7.0
API 20NE						
Reduction of nitrate to nitrite	+	−	+	+	+	−
Urease	+	−	+	+	−	−
Assimilation of						
Potassium gluconate	−	−	+	+	−	−
*N*-Acetyl-glucosamine	−	−	+	−	−	−
Malate	−	+	−	+	−	−
Trisodium citrate	+	−	−	+	−	−
API ZYM assay						
Alkaline phosphatase	+	−	−	+	+	+
Cystine arylamidase	−	−	−	+	−	−
Esterase (C4)	+	−	+	+	−	−
Esterase lipase (C8)	+	−	−	−	−	−
Leucine arylamidase	+	−	−	+	−	+
Acid phosphatase	−	−	−	−	+	−
Naphthol-AS-BI-phosphohydrolase	+	+	−	(+)	−	−
*N*-Acetyl-*β*-glucosaminidase	+	−	−	−	−	−

+, Positive; (+), weak positive reaction; −, negative; na, not available.

## Chemotaxonomy

Fatty acid methyl esters were extracted from fresh cultures of strain FBL11^T^ and analysed using the Sherlock MIDI system, in accordance with the Microbial Identification System protocol [27,29]. The fatty acid profile obtained was consistent with the characteristics of the *Psychrobacter* genus, as summarized in Table 3. Analysis of fatty acid profiles revealed that C_18:1_* ω9*c and C_17:1_* ω8*c were the dominant components in strain FBL11^T^, resembling the profiles of *P. celer* JCM 12601^T^ and *P. piechaudii* CIP110854^T^. However, *P. fulvigenes* KC-40^T^ exhibited a lower proportion of C_18:1_* ω9*c compared to FBL11^T^. C_9:0_, C_10:0_, summed feature 2 and summed feature 3 are present in FBL11^T^ and some related strains, but their specific proportions may help in differentiation, supporting its classification as a novel species.

**Table 3.** Cellular fatty acid composition (%) of strain FBL11^T^ and type strains 1, FBL11^T^; 2, *P. celer* JCM 12601^T^ [32]; 3, *P. piechaudii* CIP110854^T^ [29]; 4, *P. fulvigenes* KC-40^T^ [4]; 5, *P. coccoides* F1192^T^ [32]; 6, *P. halodurans* F2608^T^ [32]. The values represent percentages of the total fatty acid composition. Key fatty acids exceeding 10% are emphasized in bold, while those under 0.5% across all strains have been excluded. tr, traces (<0.5%); nd, not detected.

Fatty acid	1	2	3	4	5	6
Saturated						
C_9:0_	0.52	0.6	nd	tr	tr	tr
C_10:0_	3.37	1	3.8	1.8	0.9	2.5
C_11:0_	tr	0.5	0.9	tr	tr	tr
C_12:0_	nd	1.8	1.9	3.1	1.6	2.4
C_16:0_	0.73	1.9	**12.3**	1.2	1.1	1.7
C_17:0_	tr	1.3	0.7	tr	1.4	1
C_18:0_	0.81	2.4	**11.2**	2.4	3.8	4.1
C_19:0_	tr	1.3	nd	tr	1.6	1.3
Unsaturated						
C_17:1_* ω8*c	7.01	**18.9**	0.8	6.3	**22.3**	**10.4**
C_18:1_* ω9*c	**71.05**	**48.5**	**37.5**	**68.3**	**47.3**	**53.1**
C_18:3_* ω6*c (6,9,12)	0.72	tr	nd	tr	1.1	1.5
C_20:4_* ω6*,9,12,15*c*	nd	tr	1.3	tr	0.8	nd
Branched						
* *iso-C_17 : 0_	0.73	1.7	nd	0.8	4.8	1.3
* *iso-C_19 : 0_	tr	tr	nd	tr	0.7	0.9
* *iso-C_18 : 1_ h	nd	nd	nd	nd	nd	0.6
* *anteiso-C_17:0_	nd	tr	nd	tr	nd	0.7
* *anteiso-C_17:1_* ω9*c	nd	4.7	nd	tr	4.6	nd
Hydroxy						
* *C_12:0_ 3-OH	2.55	2.2	**8.2**	3.9	1.4	2.8
Summed feature*						
* *1	tr	1.8	nd	1	0.7	0.9
* *2	1.24	0.9	nd	1.9	tr	1.4
* *3	7.93	3.1	4.6	3.5	1.4	6
* *6	1.48	3.2	nd	1.4	2	1.1

* Summed features represent groups of two or three fatty acids that could not be separated using the MIDI system. Summed feature 1 comprised iso-C_15 : 1_ h and/or C_13:0_ 3-OH. Summed feature 2 comprised iso-C_16:1_ I and/or C_14:0_ 3-OH. Summed feature 3 comprised C_16:1_* ω7*c and/or C_16:1_* ω6*c. Summed feature 6 comprised C_19:1_* ω1*1c and/or C_19:1_* ω9*c.

The mass spectrum of strain FBL11^T^ was analysed using matrix-assisted laser desorption/ionization time-of-flight MS (MALDI-TOF MS). Following overnight growth on marine agar at 30 °C, a single colony was transferred to a spot on a 96-well plate. The microbial film was then overlaid with 1 µl of matrix solution (*α*-cyano-4-hydroxycinnamic acid, Bruker Daltonics) and air-dried at room temperature. The 96-well plate was inserted into the MALDI-TOF system Autoflex maX (Bruker Daltonics) for automated measurement [30]. Each spot was exposed to a pulsed nitrogen laser beam operating at 337 nm wavelength, 60 Hz frequency and 20 kV acceleration voltage. Spectra were recorded in the linear positive mode, covering the molecular weight range between 2,000 and 20,000 Da. Calibration was performed using Bruker Bacterial Test Standard *Escherichia coli* DH5α (Bruker Daltonics). Data processing was carried out using MBT Compass version 4.1.70.4 (Bruker Daltonics), and spectra were compared with the reference database for bacterial identification. However, the mass spectrum of strain FBL11^T^ did not match that of *Psychrobacter*, as the log score was below 1.7 (Table 4). Typically, a log score of 1.7 or higher is required for identification at the species or genus level [31]. The bioTyper database version 13.0 contains 23 species of *Psychrobacter* spectra (*P. aestuarii*, *Psychrobacter alimentarius*, *Psychrobacter aquaticus*, *Psychrobacter arcticus*, *Psychrobacter arenosus*, *P. celer*, *Psychrobacter ciconiae*, *Psychrobacter faecalis*, *Psychrobacter frigidicola*, *P. fulvigenes*, *P. immoblis*, *Psychrobacter jeotgali*, *Psychrobacter luti*, *Psychrobacter lutiphocae*, *Psychrobacter maritimus*, *Psychrobacter namhaensis*, *Psychrobacter pacificensis*, *Psychrobacter pasteurii*, *Psychrobacter phenylpyruvicus*, *P. piechaudii*, *Psychrobacter pulmonis*, *Psychrobacter sanguinis* and *Psychrobacter vallis*).

**Table 4.** Identification of *Psychrobacter* species using MALDI-TOF MS with corresponding score values

Sample ID	Organism (best match)	Score value
FBL11^T^	No organism identification possible	1.43
*Psychrobacter aquimaris* KACC 17271^T^	No organism identification possible	1.56
*Psychrobacter immobilis* KACC 15294^T^	*Psychrobacter immobilis*	2.09
*P. jeotgali* KACC 17235^T^	*P. jeotgali*	2.19
*P. pacificensis* KACC 15295^T^	No organism identification possible	1.43
*P. namhaensis* KACC 17272^T^	*P. namhaensis*	2.20

Interpretation of score values: ≥2.00, secure species identification; 1.70–1.99, probable genus identification; <1.70, no reliable identification possible.

## Description of *Psychrobacter saeujeotis* sp. nov.

*Psychrobacter saeujeotis* (sae.u.jeo’tis. L. n. *saeu*, shrimp in Korean; L. n. *jeot*, a traditional Korean fermented seafood; N.L. gen. n. *saeujeotis*, of shrimp jeotgal).

Cells are Gram-negative, non-motile and strictly aerobic. On marine agar plates incubated at 30 °C for 2 days, colonies grow to 3–5 mm in diameter, displaying a smooth, wet, circular and translucent beige appearance. Optimal growth occurs at 20–30 °C with NaCl concentrations ranging from 0 to 15% and at a pH of 7.0. The genome size is 3,294,493 bp with a DNA G+C content of 42.5 mol%. This strain exhibits positive oxidase activity, hydrolyses urea and reduces nitrate to nitrite. It utilizes trisodium citrate as a carbon source but does not produce acetoin or hydrogen sulphide, nor does it hydrolyse gelatin. The type strain, FBL11^T^ (=KACC 23745^T^=KCTC 8655^T^=JCM 37231^T^), was originally isolated from salted shrimp jeotgal. GenBank accession numbers for the 16S rRNA gene sequence and genome sequence of the type strain are PQ498527 and JBDGHN01, respectively.

## Supplementary material

10.1099/ijsem.0.006734 Uncited Supplementary Material 1.

## Abbreviations

ANI: average nucleotide identity
BIC: Bayesian information criterion
dDDH: digital DNA–DNA hybridization
NA: nutrient agar
NCBI: National Center for Biotechnology Information
TYGS: Type Strain Genome Server
