# Supplementary material for: Psychrobacter saeujeotis sp. nov., a novel halophilic bacterium isolated from salted shrimp jeotgal
Source: Int J Syst Evol Microbiol. 2025 Mar 21;75(3):006734. doi: 10.1099/ijsem.0.006734 (PMC12281858; doi:10.1099/ijsem.0.006734)
Supplement: Uncited Supplementary Material 1. [file ijsem-75-06734-s001.pdf]

Supplementary Materials

Table S1. Validly published and correct name of the genus *Psychrobacter*

Name	Nomenclatural status	Taxonomic status
Psychrobacter adeliensis Shivaji et al. 2005	Validly published under the ICNP	Correct name
Psychrobacter aestuarii Baik et al. 2010	Validly published under the ICNP	Correct name
Psychrobacter alimentarius Yoon et al. 2005	Validly published under the ICNP	Correct name
Psychrobacter aquaticus Shivaji et al. 2005	Validly published under the ICNP	Correct name
Psychrobacter aquimaris Yoon et al. 2005	Validly published under the ICNP	Correct name
Psychrobacter arcticus Bakermans et al. 2006	Validly published under the ICNP	Correct name
Psychrobacter arenosus Romanenko et al. 2004	Validly published under the ICNP	Correct name
Psychrobacter celer Yoon et al. 2005	Validly published under the ICNP	Correct name
Psychrobacter cibarius Jung et al. 2005	Validly published under the ICNP	Correct name
Psychrobacter ciconiae Kämpfer et al. 2015	Validly published under the ICNP	Correct name
Psychrobacter coccoides Shang et al. 2022	Validly published under the ICNP	Correct name
Psychrobacter communis **Pallen 2024**	**Validly published under the ICNP**	**Correct name**
Psychrobacter cryohalolentis Bakermans et al. 2006	Validly published under the ICNP	Correct name
Psychrobacter faecalis Kämpfer et al. 2002	Validly published under the ICNP	Correct name
Psychrobacter fjordensis Zeng et al. 2022	Validly published under the ICNP	Correct name
Psychrobacter fozii Bozal et al. 2003	Validly published under the ICNP	Correct name
Psychrobacter frigidicola Bowman et al. 1996	Validly published under the ICNP	Correct name
Psychrobacter fulvigenes Romanenko et al. 2009	Validly published under the ICNP	Correct name
Psychrobacter glaciei Zeng et al. 2016	Validly published under the ICNP	Correct name
Psychrobacter glacincola Bowman et al. 1997	Validly published under the ICNP	Correct name
Psychrobacter halodurans Shang et al. 2022	Validly published under the ICNP	Correct name
Psychrobacter immobilis Juni and Heym 1986	Validly published under the ICNP	Correct name
Psychrobacter jeotgali Yoon et al. 2003	Validly published under the ICNP	Correct name
Psychrobacter luti Bozal et al. 2003	Validly published under the ICNP	Correct name
Psychrobacter lutiphocae Yassin and Busse 2009	Validly published under the ICNP	Correct name
Psychrobacter marincola Romanenko et al. 2002	Validly published under the ICNP	Correct name
Psychrobacter maritimus Romanenko et al. 2004	Validly published under the ICNP	Correct name
Psychrobacter namhaensis Yoon et al. 2005	Validly published under the ICNP	Correct name
Psychrobacter nivimaris Heuchert et al. 2004	Validly published under the ICNP	Correct name

Psychrobacter oceani Matsuyama et al. 2015	Validly published under the ICNP	Correct name
Psychrobacter okhotskensis Yumoto et al. 2003	Validly published under the ICNP	Correct name
Psychrobacter pacificensis Maruyama et al. 2000	Validly published under the ICNP	Correct name
Psychrobacter pasteurii Hurtado-Ortiz et al. 2017	Validly published under the ICNP	Correct name
Psychrobacter phenylpyruvicus (Bøvre and Henriksen 1967) Bowman et al. 1996	Validly published under the ICNP	Correct name
Psychrobacter piechaudii Hurtado-Ortiz et al. 2017	Validly published under the ICNP	Correct name
Psychrobacter piscatorii Yumoto et al. 2010	Validly published under the ICNP	Correct name
Psychrobacter pocilloporae Zachariah et al. 2016	Validly published under the ICNP	Correct name
Psychrobacter proteolyticus Denner et al. 2001	Validly published under the ICNP	Correct name
Psychrobacter pulmonis Vela et al. 2003	Validly published under the ICNP	Correct name
Psychrobacter pygoscelis Kämpfer et al. 2020	Validly published under the ICNP	Correct name
Psychrobacter raelei Manzulli et al. 2024	Validly published under the ICNP	Correct name
Psychrobacter salsus Shivaji et al. 2005	Validly published under the ICNP	Correct name
Psychrobacter sanguinis Wirth et al. 2012	Validly published under the ICNP	Correct name
Psychrobacter submarinus Romanenko et al. 2002	Validly published under the ICNP	Correct name
Psychrobacter urativorans Bowman et al. 1996	Validly published under the ICNP	Correct name
Psychrobacter vallis Shivaji et al. 2005	Validly published under the ICNP	Correct name

Bold type, 16S rRNA gene sequence was not reported, so it was excluded from the phylogenetic analysis based on the 16S rRNA gene.

Table S2. 16S rRNA gene sequence information for *Psychrobacter* type strains

Species	Strain	Accession no.	Nucleotide size (bp)
Moraxella lacunata	ATCC 17967	D64049	1519
Psychrobacter adeliensis	DSM 15333	NR_117634.1	1526
Psychrobacter aestuarii	SC 35	EU939718	1424
Psychrobacter alimentarius	JG-100	AY513645	1494
Psychrobacter aquaticus	CMS 56	AJ584833	1481
Psychrobacter aquimaris	SW-210	AY722804	1494
Psychrobacter arcticus	273-4	AY444822	1504
Psychrobacter arenosus	R-7	AJ609273	1491
Psychrobacter celer	SW-238	AY842259	1495
Psychrobacter cibarius	JG-219	AY639871	1494
Psychrobacter ciconiae	176/10	KM486054	1417
Psychrobacter coccoides	F1192	MW405808	1503
Psychrobacter cryohalolentis	K5	AY660685	1500
Psychrobacter faecalis	Iso-46	AJ421528	1496
Psychrobacter fjordensis	BSw21516B	GQ358940	1500
Psychrobacter fozii	strain NF23	AJ430827	1491
Psychrobacter frigidicola	DSM 12411	AJ609556	1528
Psychrobacter fulvigenes	KC 40	AB438958	1532
Psychrobacter glaciei	BIc20019	FJ748508	1500
Psychrobacter glacincola	DSM 12194	AJ312213	1515
Psychrobacter halodurans	F2608	MW405795	1503
Psychrobacter immobilis	DSM 7229	AJ309942	1492
Psychrobacter jeotgali	YKJ-103	AF441201	1494
Psychrobacter luti	strain NF11	AJ430828	1491
Psychrobacter lutiphocae	IMMIB L-1110	FM165580	1502
Psychrobacter marincola	KMM 277	AJ309941	1428
Psychrobacter maritimus	Pi2-20	AJ609272	1476
Psychrobacter namhaensis	SW-242	AY722805	1494
Psychrobacter nivimaris	1988-02-07	AJ313425	1498
Psychrobacter oceani	4k5	AB910522	1465
Psychrobacter okhotskensis	MD17	AB094794	1492
Psychrobacter pacificensis	NIBH P2K6	AB016057	1526
Psychrobacter pasteurii	CIP 110853	KY292376	1460
Psychrobacter phenylpyruvicus	NBRC 102152	AB681720	1460
Psychrobacter piechaudii	CIP 110854	KY292375	1446
Psychrobacter piscatorii	T-3-2	AB453700	1510
Psychrobacter pocilloporae	S6-60	KT444699	1405
Psychrobacter proteolyticus	116	AJ272303	1426
Psychrobacter pulmonis	CECT 5989	AJ437696	1363
Psychrobacter pygoscelis	I-STPP5b	MH065724	1448
Psychrobacter salsus	DD 48	AJ539104	1454
Psychrobacter sanguinis	13983	HM212668	1457
Psychrobacter submarinus	KMM 225	AJ309940	1428
Psychrobacter urativorans	DSM 14009	AJ609555	1529
Psychrobacter vallis	CMS 39	AJ584832	1481
Psychrobacter saeujeotis	FBL11	PQ498527	1454

Psychrobacter raelei	PraFG1	MK771149	1425

Table S3. Model selection statistics for phylogenetic inference

Model	LogL	AIC	AICc	BIC
TPM3+I+R2	-6888.459	13968.919	13981.719	14482.26
TVMe+I+R2	-6881.687	13959.375	13972.729	14483.41
TVM+F+I+R2	-6871.234	13944.467	13958.677	14484.545
TVM+F+R3	-6868.549	13941.099	13955.6	14486.523

LogL (Log-Likelihood): The logarithm of the likelihood score for the model. Higher values (closer to zero) indicate a better fit to the data.
AIC (Akaike Information Criterion): A criterion used for model selection where lower values indicate a better balance between model fit and complexity.
AICc (Corrected Akaike Information Criterion): AIC adjusted for small sample sizes, providing more accurate model selection in cases with limited data.
BIC (Bayesian Information Criterion): A criterion for model selection that penalizes model complexity more heavily than AIC. Lower BIC values indicate a better model fit considering both the model's goodness-of-fit and its complexity.

Table S4. Summary of genome features of the genus *Psychrobacter*

#Organism Name	Strain	Size (Mb)	GC%	CDS	Level	Assembly no.
Psychrobacter adeliensis	SJ 14	3.1	43.0	2496	Contig	GCF_904845895.1
Psychrobacter aestuarii	SC 35	2.8	49.5	2321	Contig	GCF_904846035.1
Psychrobacter alimentarius	JG 100	3.4	43.0	2780	Contig	GCF_904845935.1
Psychrobacter aquaticus	CMS 56	3.2	43.0	2663	Contig	GCF_000471625.1
Psychrobacter aquimaris	DSM 16329	3.4	43.0	2889	Contig	GCF_016107525.1
Psychrobacter arcticus	273-4	2.7	43.0	2138	Complete	GCF_000012305.1
Psychrobacter arenosus	R7	3.7	45.0	2862	Complete	GCF_904848165.1
Psychrobacter celer	DSM 23510	3.0	46.5	2441	Contig	GCF_016107555.1
Psychrobacter cibarius	DSM 16327	3.2	43.0	2661	Contig	GCF_016107535.1
Psychrobacter ciconiae	176-10	2.5	45.5	2134	Contig	GCF_904846055.1
Psychrobacter coccoides	F1192	3.1	45.0	2546	Scaffold	GCF_017498085.1
Psychrobacter communis	Sa4CVA2	2.9	43.5	2395	Scaffold	GCF_014836505.1
Psychrobacter cryohalolentis	K5	3.1	42.0	2518	Complete	GCF_000013905.1
Psychrobacter faecalis	Iso-46	3.1	43.5	2523	Contig	GCF_904845915.1
Psychrobacter fjordensis	BSw21516B	3.4	42.5	2739	Contig	GCF_904845995.1
Psychrobacter fozii	CECT 5889	3.5	42.5	2839	Scaffold	GCF_003217155.1
Psychrobacter frigidicola	ACAM 304	2.8	42.0	2343	Contig	GCF_007997305.1
Psychrobacter fulvigenes	KC-40	3.5	44.0	2829	Contig	GCF_904846155.1
Psychrobacter glaciei	KCTC 42280	3.4	43.5	2749	Scaffold	GCF_014652895.1
Psychrobacter glacincola	ACAM483	3.2	42.5	2629	Contig	GCF_904846215.1
Psychrobacter halodurans	F2608	2.9	47.5	2333	Contig	GCF_017498075.1
Psychrobacter immobilis	DSM 7229	3.2	43.0	2639	Scaffold	GCF_003148585.1
Psychrobacter jeotgali	YKJ-103	3.1	42.5	2566	Contig	GCF_904846315.1
Psychrobacter luti	CECT 5885	3.0	42.5	2407	Scaffold	GCF_014192115.1
Psychrobacter lutiphocae	DSM 21542	3.2	41.5	2548	Scaffold	GCF_000382145.1
Psychrobacter marincola	KMM 277	3.1	43.0	2503	Contig	GCF_904846325.1
Psychrobacter maritimus	Pi2-20	3.2	43.0	2561	Contig	GCF_904846295.1
Psychrobacter namhaensis	DSM 16330	2.9	45.0	2376	Contig	GCF_016107545.1
Psychrobacter nivimaris	1988-02-07	3.4	43.0	2794	Contig	GCF_904846365.1

Psychrobacter oceani	4k5	3.0	44.0	2479	Contig	GCF_904846375.1
Psychrobacter okhotskensis	MD 17	3.4	43.5	2808	Contig	GCF_904846405.1
Psychrobacter pacificensis	NBRC 103191	3.1	44.0	2633	Scaffold	GCF_030160475.1
Psychrobacter pasteurii	CIP 110853	2.9	42.5	2507	Contig	GCF_900162815.1
Psychrobacter phenylpyruvicus	DSM 7000	3.1	41.5	2548	Scaffold	GCF_000685805.1
Psychrobacter piechaudii	CIP 110854	2.8	42.5	2386	Scaffold	GCF_900162825.1
Psychrobacter piscatorii	T-3-2	3.5	43.5	2928	Contig	GCF_904846415.1
Psychrobacter pocilloporae	S6-60	3.1	44.0	2563	Contig	GCF_029872915.1
Psychrobacter proteolyticus	116	3.0	43.0	2482	Contig	GCF_904846455.1
Psychrobacter pulmonis	S-606	3.0	43.5	2407	Contig	GCF_904846465.1
Psychrobacter pygoscelis	I-STPP5b	3.4	44.5	2711	Contig	GCF_004335015.1
Psychrobacter raelei	PraFG1	3.2	44.5	2536	Complete	GCF_022631235.3
Psychrobacter saeujeotis	FBL11	3.3	42.5	2864	Contig	GCF_039615135.1
Psychrobacter salsus	DD48	2.9	45.0	2392	Contig	GCF_904846445.1
Psychrobacter sanguinis	13983	3.2	41.5	2543	Contig	GCF_904846515.1
Psychrobacter submarinus	KMM 225	3.0	45.0	2505	Contig	GCF_904846685.1
Psychrobacter urativorans	ACAM534	3.5	41.5	2779	Contig	GCF_904846695.1
Psychrobacter vallis	CMS 39	3.2	43.5	2642	Contig	GCF_904846715.1

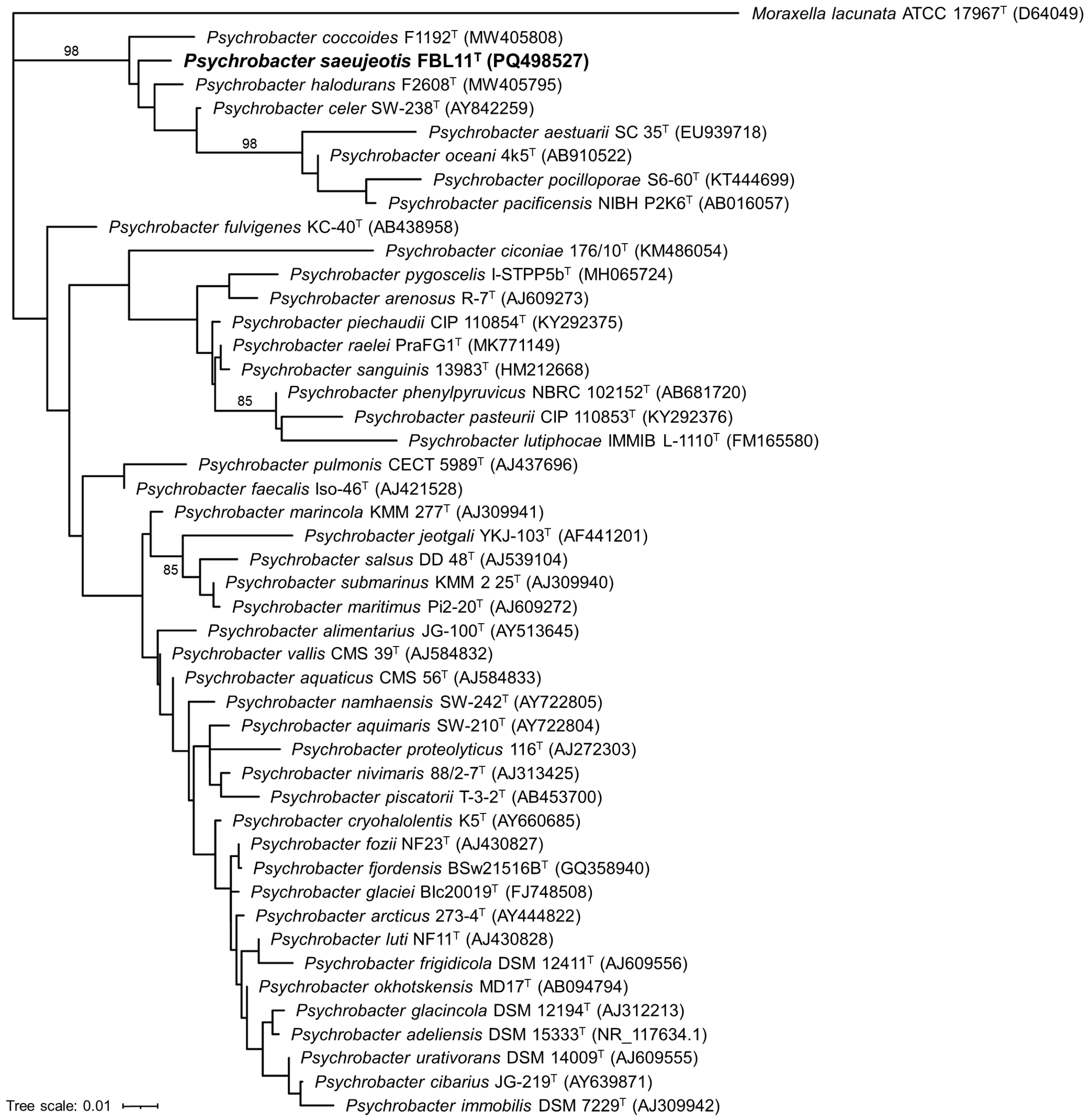

Fig. S1. Neighbor-joining tree showing the phylogenetic relationship of strain FBL11^{T} and type strains of all species within the genus *Psychrobacter* based on nucleotide sequences from the 16S rRNA gene. *Moraxella lacunata* was used as the outgroup. Bootstrap values (based on 1000 replicates) higher than 80% are shown at nodes. The species from this study is indicated in bold. The scale bar indicates 1% substitutions per site.

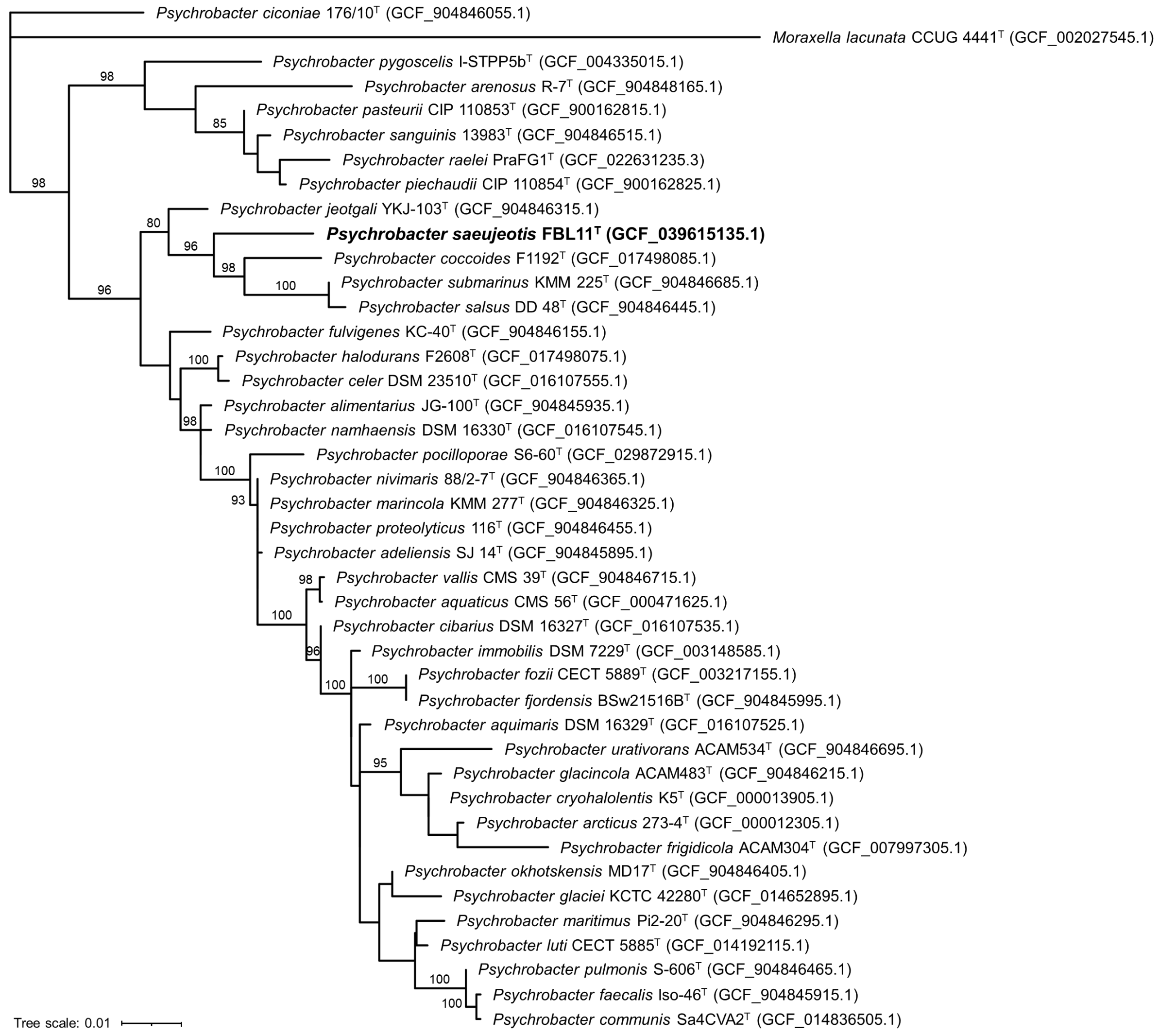

Fig. S2. Maximum-likelihood phylogenetic tree based on 23S rRNA gene sequences, reconstructed using the GTR+F+R2 evolutionary model, illustrating the relationships between strain FBL11^{T} and other *Psychrobacter* species. *Moraxella lacunata* was used as the outgroup. Bootstrap values over 80% (from 1000 replicates) are shown at each node, with the species from this study marked in bold. The scale bar indicates 1% substitutions per site.

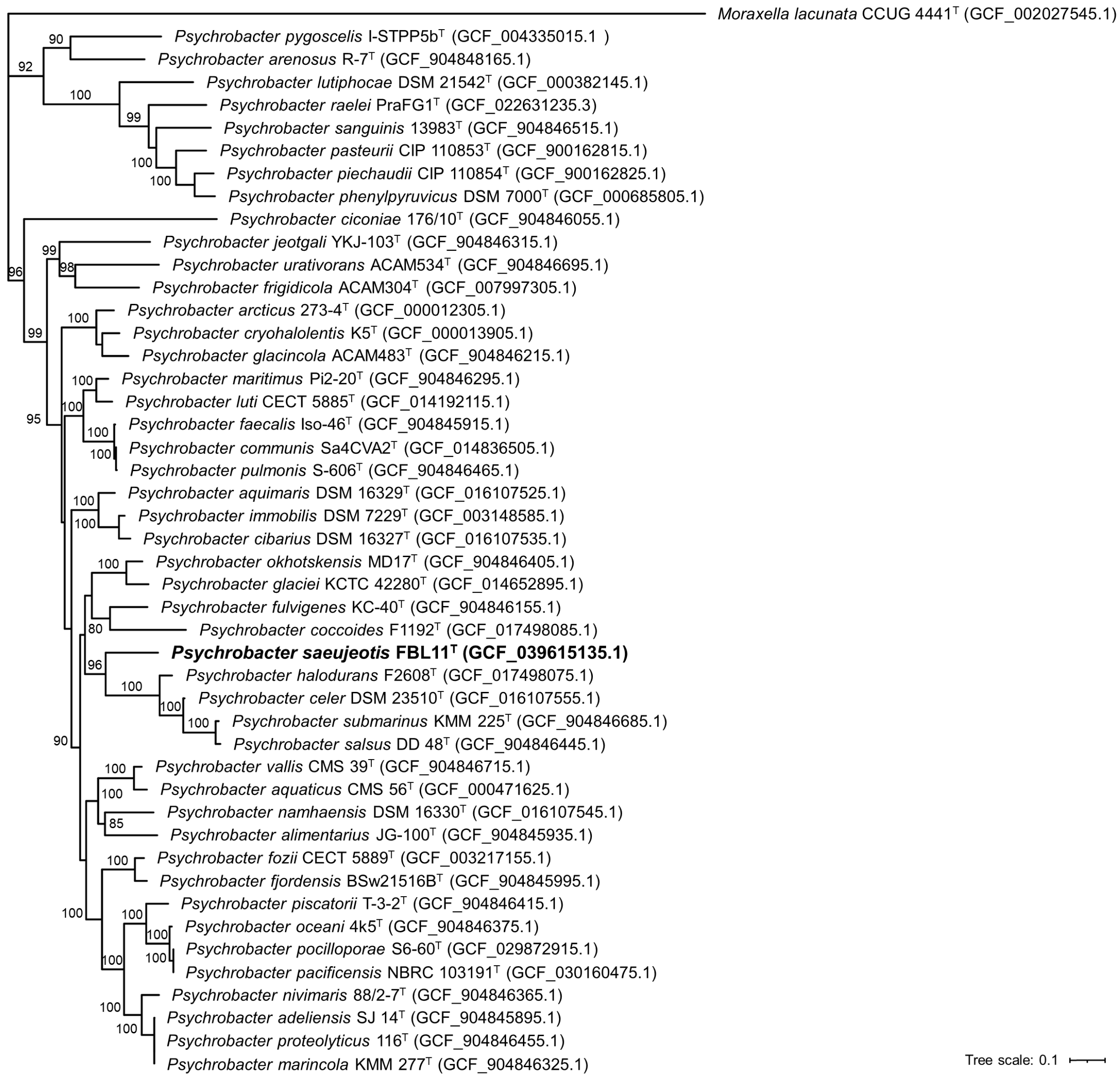

Fig. S3. Maximum-likelihood phylogenetic tree based on *gyrB* gene, reconstructed using the TNe+I+R4 evolutionary model, illustrating the relationships between strain FBL11^T and other *Psychrobacter* species. *Moraxella lacunata* was used as the outgroup. Bootstrap values over 80% (from 1000 replicates) are shown at each node, with the species from this study marked in bold. The scale bar indicates 1% substitutions per site.
